# Prenatal and postnatal traffic pollution exposure, DNA methylation in *Shank3* and *MeCP2* promoter regions, H3K4me3 and H3K27me3 and sociability in rats’ offspring

**DOI:** 10.1186/s13148-021-01170-x

**Published:** 2021-09-26

**Authors:** Qinfeng Zhou, Yu Tian, Chenlu Xu, Juling Wang, Yongtang Jin

**Affiliations:** 1 Environmental Epigenetics Laboratory, Department of Environmental Medicine, School of Public Health, Zhejiang University, 866 Yuhangtang Rd, Hangzhou, 310058 Zhejiang Province People’s Republic of China; 2 Department of General Practice, Sir Run Run Shaw Hospital, Medical College of Zhejiang University, Hangzhou, Zhejiang Province People’s Republic of China

**Keywords:** Traffic pollution exposure, DNA methylation, MeCP2, Shank3, H3K4me3, H3K27me3, Sociability

## Abstract

**Background:**

Road traffic air pollution is linked with an increased risk of autistic spectrum disorder (ASD). The aim of this study is to assess the effect of exposure to prenatal or postnatal traffic-related air pollution combining concomitant noise pollution on ASD-related epigenetic and behavioral alternations on offspring.

**Methods:**

A 2 × 2 factorial analysis experiment was designed. Wistar rats were exposed at different sites (L group: green space; H group: crossroads) and timings (E group: full gestation; P group: 21 days after birth) at the same time, and air pollutants of nitrogen dioxide (NO_2_) and fine particles (PM_2.5_) were meanwhile sampled. On postnatal day 25, brains from offspring of each group were extracted to determine the levels of DNA methylation in *Shank3* (three parts: *Shank3_01*, *Shank3_02*, *Shank3_03*) and *MeCP2* (two parts: *MeCP2_01*, *MeCP2_02*) promoter regions, H3K4me3 and H3K27me3 after three-chamber social test. Meanwhile, the Shank3 and MeCP2 levels were quantified.

**Results:**

The concentrations of PM_2.5_ (L: 58.33 µg/m^3^; H: 88.33 µg/m^3^, *P* < 0.05) and NO_2_ (L: 52.76 µg/m^3^; H: 146.03 µg/m^3^, *P* < 0.01) as well as the intensity of noise pollution (L: 44.4 dB (A); H: 70.1 dB (A), *P* < 0.001) differed significantly from 18:00 to 19:00 between experimental sites. Traffic pollution exposure (*P* = 0.006) and neonatal exposure (*P* = 0.001) led to lower weight of male pups on PND25. Male rats under early-life exposure had increased levels of *Shank3* (*Shank3_02*: timing *P* < 0.001; site *P* < 0.05, *Shank3_03*: timing *P* < 0.001) and *MeCP2* (*MeCP2_01*: timing *P* < 0.001, *MeCP2_02*: timing *P* < 0.001) methylation and H3K4me3 (EL: 11.94 µg/mg; EH: 11.98; PL: 17.14; PH: 14.78, timing *P* < 0.05), and reduced levels of H3K27me3 (EL: 71.07 µg/mg; EH: 44.76; PL: 29.15; PH: 28.67, timing *P* < 0.001; site *P* < 0.05) in brain compared to those under prenatal exposure. There was, for female pups, a same pattern of *Shank3* (*Shank3_02*: timing *P* < 0.001; site *P* < 0.05, *Shank3_03*: timing *P* < 0.001) and *MeCP2* (*MeCP2_01*: timing *P* < 0.05, *MeCP2_02*: timing *P* < 0.001) methylation and H3K4me3 (EL: 11.27 µg/mg; EH: 11.55; PL: 16.11; PH: 15.44, timing *P* < 0.001), but the levels of H3K27me3 exhibited an inverse trend concerning exposure timing. Hypermethylation at the *MeCP2* and *Shank3* promoter was correlated with the less content of MeCP2 (female: EL: 32.23 ng/mg; EH: 29.58; PL: 25.01; PH: 23.03, timing *P* < 0.001; site *P* < 0.05; male: EL: 31.05 ng/mg; EH: 32.75; PL: 23.40; PH: 25.91, timing *P* < 0.001) and Shank3 (female: EL: 5.10 ng/mg; EH: 5.31; PL: 4.63; PH: 4.82, timing *P* < 0.001; male: EL: 5.40 ng/mg; EH: 5.48; PL: 4.82; PH: 4.87, timing *P* < 0.001). Rats with traffic pollution exposure showed aberrant sociability preference and social novelty, while those without it behaved normally.

**Conclusions:**

Our findings suggest early life under environmental risks is a crucial window for epigenetic perturbations and then abnormalities in protein expression, and traffic pollution impairs behaviors either during pregnancy or after birth.

**Supplementary Information:**

The online version contains supplementary material available at 10.1186/s13148-021-01170-x.

## Introduction

Autistic spectrum disorder (ASD) is a neurodevelopmental disturbance characterized by common deficits in social interaction, the ability to communicate and repetitive patterns of behavior [1]. There is a non-consensus over the ASD onset [2]. Although genetic alterations are frequently observed, the cause in most ASDs remains elusive, and only approximately 25% of clinical ASD diagnoses can be accounted for by genetic sequencing analyses [3]. Estimated 40–50% of variance in ASD liability can be attributed to environmental factors, such as air pollution, parental age and nutrition [4].

Traffic pollution, which is an important source of ambient air and noise pollution, is implicated in ASD [5]. Predominant epidemiologic studies focus on prenatal air pollution exposure contributing to an increased risk of ASD, particularly of the first and third trimester [6–8], while early-life exposure seems to be associated with it [9]. Moreover, mice that were exposed from embryonic day 0 to postnatal day 21 to diesel exhaust exhibited autism-like behavioral changes [10]. A comparison drawn between maternal exposure and neonatal one has yet to be investigated in animals.

The developing central nervous system in prenatal and early postnatal phases is particularly vulnerable to environmental insults that can lead to epigenetic perturbations [11]. DNA methylation and histone posttranslational modifications (HPTMs) as epigenetic biomarkers regulate gene expression and ultimately cellular function in the central nervous system (CNS). Also, disturbance of these epigenetic mechanisms can translate early external insults into long-lasting brain damage [12]. Moreover, animal studies to determine epigenetic alterations are needed due to difficult access to postmortem brains of ASD cases, postmortem influences on DNA methylation and the discordance between peripheral tissues like blood and brain.

SH3 and multiple ankyrin repeat domains 3 (Shank3) are postsynaptic scaffolding protein that interacts with various synaptic molecules. It functions in targeting, anchoring and regulating postsynaptic neurotransmitter receptors and signaling molecules [13, 14]. Deficits in synaptic maturation, connectivity or stabilization represent a core neuropathological mechanism, which is likely to underlie the etiology of ASD [15]. Previous studies have consistently demonstrated that the loss of Shank3 in mice resulted in hallmarks of ASD, including repetitive behavior and social interaction deficits [16].

Methyl-CpG-binding protein 2 (MeCP2) can interact with DNA and specifically bind to 5mC and 5hmC and then regulate the expression of genes in a gene-length-associated manner [17]. MeCP2 plays an important role in neuronal differentiation, synaptogenesis and chromatin structure establishment and maintenance [18], and its mutations cause Rett syndrome (RTT) showing ASD-like behaviors [19, 20]. A significant increase in *MeCP2* promoter methylation in frontal cortex and a correlated reduction in *MeCP2* protein expression were observed in ASD patients [21].

Tri-methylation of lysine 27 on histone H3 (H3K27me3) has long been linked to transcriptional repression, whereas tri-methylation of lysine 4 on histone H3 (H3K4me3) has an opposite effect that activates gene expression [22, 23]. Aberrant regulation of the histone methylation can influence the expression of developmental genes and then contribute to the pathogenesis of various CNS disorders [24].

Based on that, we hypothesize that a certain window of traffic pollution exposure can lead to deviations of the methylation and protein expression of MeCP2 and Shank3, H3K4me3 and H3K27me3, which ultimately impairs normal social interaction. Accordingly, we designed a 2 × 2 factorial and real-world experiment that rats were , respectively, exposed to two sites (green space vs. crossroads) and different timings (entire gestation vs. three weeks after birth) to investigate the impact of discrete traffic-related air pollution combining simultaneous noise pollution on ASD-related epigenetic and behavioral alternations.

## Methods

### Animals and experimental process

**Fig. 1:**
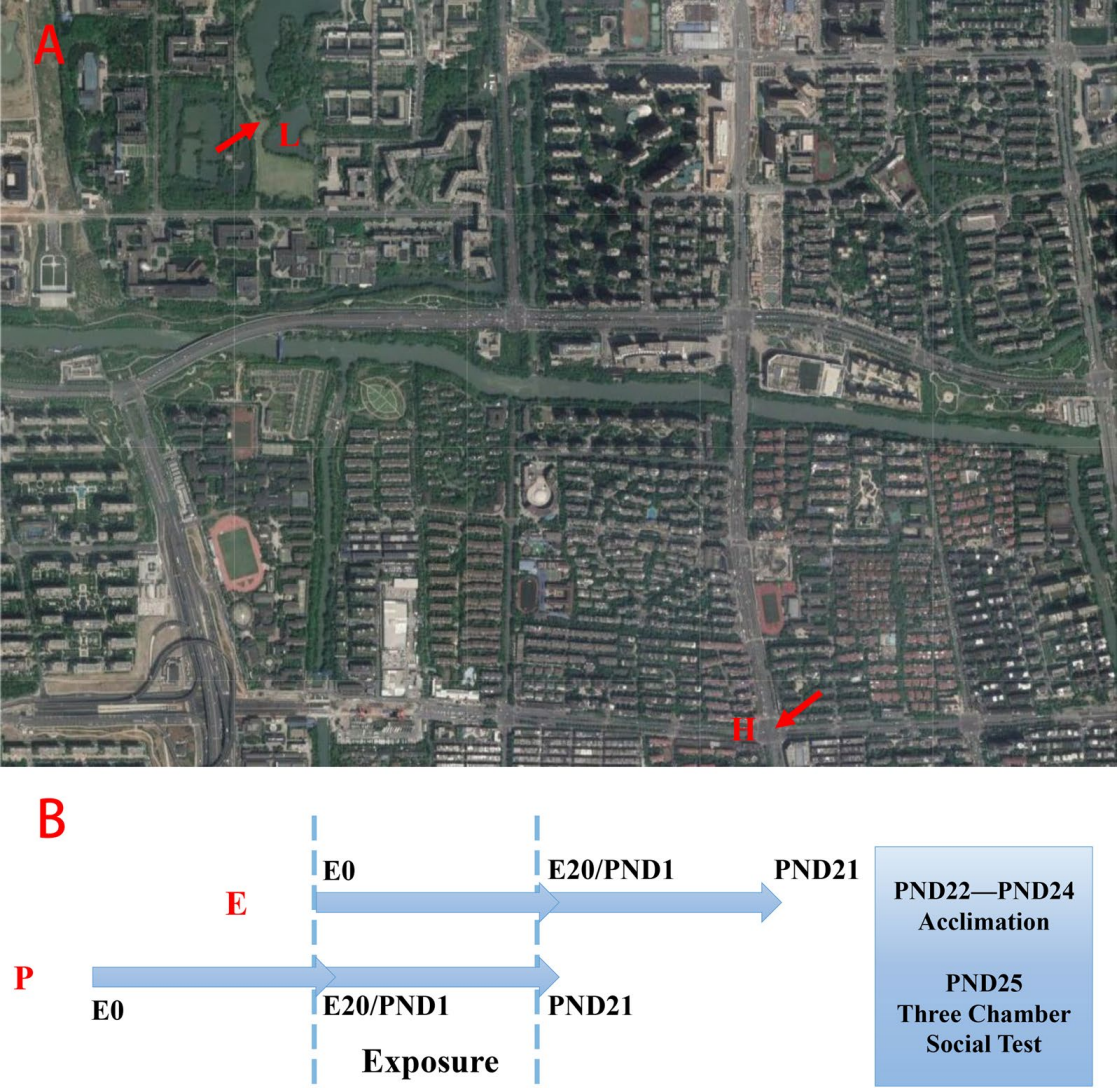
Exposure sites and study design. **A** Map of exposure places. L: green space; H: intersection **B** Experimental design. E: exposure from embryonic day 0 (E0) to embryonic day 20 (E20); P: exposure from postnatal day (PND) 1 to postnatal day 21

Thirty-six pairs of Wistar rats (specific pathogen-free, SPF) aged eight weeks old were adopted from the Laboratory Animal Center, Zhejiang University and then were housed in a room providing a 12-h light/dark cycle and HEPA-filtered air via an air circulation system. All rats were, respectively, placed into acrylic cages with free access to food and water and acclimated to the environment for one week prior to co-housing. Monogamously pregnant dams (E group) in half were exposed from embryonic day 0 (E0) to embryonic day 20 (E20) at a green space (L group) that is adjacent to a lake and in a wood, or a junction (H group) which connecting both four-lane roads is more than 3500 vehicles per hour at peak traffic intensity, while the others with litters (P group) experienced the same exposure from postnatal day (PND) 1 to postnatal day 21. Thus, there were four groups with 9 pairs each group, respectively, named EL, EH, PL and PH, and the procedures are diagramed in Fig. 1. Exposure lasted for 12 h per day (07:30–19:30), which was identical with the light condition in the room. Offspring were culled to 5 or 6 per cage, particularly ones with weakness and low birth weight. From all pups after the experiment, ten healthy and randomly chosen offspring of both sexes of each group experienced a three-day acclimation in the Laboratory Animal Center, Zhejiang University, after termination of exposure and weaning (PND21). On PND25, all the offspring were performed under three-chamber social test and instantly measured weight and then killed. Extracted brains were snap-frozen in liquid nitrogen immediately and stored at − 80 ℃. All procedures involving animals were approved by the Institutional Animal Care and Use Committee of Zhejiang University and completely finished between October and November 2018.

### Traffic-related air pollution and noise measurement

Particulate matter with aerodynamic diameter < 2.5 µm (PM_2.5_) was collected using a medium volume filter sampler at the speeds of 100 L per minute (Hengda, Hangzhou, China). In the meanwhile, nitrogen dioxide (NO_2_) was sampled (Sirui, Jiangsu, China). Traffic noise was recorded by a hand-held sound meter (Aihua, Hangzhou, China). All data were harvested during rush hour (18:00–19:00).

### DNA and histone extraction

DNA of the frontal cortex separated from the left brain was isolated with Rapid Animal Genomic DNA Isolation Kit (Sangon Biotech, Shanghai, China). Histone was extracted from the right brain by EpiQuik™ Total Histone Extraction Kit (Epigentek, NY, USA), with quantification using the BCA Protein Assay Kit (Beyotime, Shanghai, China). All procedures were in accordance with the manufacturer’s instructions.

### DNA and histone methylation analyses and protein expression

CpG islands in close proximity to promoter regions of *MeCP2* and *Shank3* follow inclusion criteria: (1) regions within a window of 2000/ + 1000 bp relative to the putative TSS locations; (2) observed/expected dinucleotide ratio ≥ 0.6; (3) length > 200 bp; (4) guanine–cytosine content ≥50% [25]. Therefore, there are 5 regions of target CpG islands (*MeCP2_01*, *MeCP2_02*, *Shank3_03*, *Shank3_04*, *Shank3_05*) and corresponding 12, 26, 14, 20 and 26 CpG sites. (Details are listed in Table 1 and Additional file 1: Table S1.)

**Table 1:** Details of the CpG regions in the CpG islands of *MeCP2* and *Shank3*

Target	Chr	TSS	Start	End	Length	Target strand	Distance 2TSS
*MeCP2_01*	X	156650388	156650143	156649855	289	–	− 245
*MeCP2_02*	X	156650388	156650143	156649855	267	–	539
*Shank3_01*	7	130474287	130473474	130473279	196	–	− 813
*Shank3_02*	7	130474287	130473474	130473279	199	–	697
*Shank3_03*	7	130474287	130473474	130473279	252	–	557

Chr: chromosome; TSS: transcription start site; Start/End: start/end position on the reference genome; Target strand: the product orientation; Distance 2TSS: the distance from the product to the TSS

Quantitative DNA methylation levels by using MethylTarget™ (Genesky Biotech, Shanghai, China) were calculated, which has been widely published [26, 27]. EZ DNA Methylation-Gold Kit (Zymo, CA, USA) was utilized to convert all unmethylated cytosine to uracil. The samples were then sequenced on an Illumina HiSeq Sequencer (CA, USA) following polymerase chain reactions (PCRs) to amplify the target DNA sequences. The mean methylation levels were computed by software Biq-analyzer.

EpiQuik™ Global Tri-Methyl Histone H3-K4/H3-K27 Quantification Kit (Epigentek, NY, USA) was employed based on colorimetric assay to determine the amount of H3K4me3 and H3K27me3. Rat MeCP2/Shank3 ELISA kit (ELASA LAB, Wuhan, China; Bioswamp, Wuhan, China, respectively) was used to quantify the content of MeCP2 and Shank3.

### Three-chamber social test

The apparatus (626 cm long × 410 cm wide × 200 cm tall) consists of an empty middle chamber and two end compartments divided by a partition where a door (195 cm long × 100 cm wide) allows rats free access to each part. All chambers were thoroughly cleaned with 75% ethanol after each test.

In the first adaptation phase, the test rat was placed in the middle chamber with two doors open and then freely explored the apparatus for 5 min. Following was the sociability phase. A strange rat (sex, strain and age-matched) was randomly placed into a mesh cylinder in an end chamber, while a same cylinder without rat was put in the opposite end chamber. The test rat was then allowed to explore chambers for 10 min. The total amount of time spent in interaction with the novel rat and the empty cage was recorded (Stoelting, Illinois, USA). Finally, in the social novelty phase, another unfamiliar rat (sex, strain and age-matched) was placed into the previously empty cage, and other conditions remained. Duration of interacting the new rat or the previous one was analyzed.

### Statistical analyses

All data were tested for normality using the Kolmogorov–Smirnov test. To investigate the influences of exposure timing and traffic pollution exposure, as well as the interaction between them on rats’ weight, the levels of *MeCP2* and *Shank3* methylation and their protein expression, H3K4me3 and H3K27me3, analysis of variance (ANOVA) with a 2 (E or P) × 2 (L or H) factorial design were performed. Simple effect analysis was further conducted if there was an interaction to determine differences at a certain level, but otherwise a non-interaction model was used to calculate the main effect of every variable. Comparisons between traffic pollutants at different places or the times obtained from the behavioral experiment were drawn by Student’s *t*-test or nonparametric Mann–Whitney test. These analyses mentioned above were processed on SPSS software (SPSS20.0). The figures showing the percent of *MeCP2* and *Shank3* methylation were achieved on R (version 3.1.2).

Data in normal distribution were presented as mean ± standard deviation (SD) and otherwise as median with 25th and 75th percentile. The threshold of significance was 0.05 during testing.

## Results

### Traffic-related air and noise pollution

Table 2 shows the levels of PM_2.5_, NO_2_ and noise from 18:00 to 19:00 at two exposure sites, a green space and a crossroads. Between them, there were significant differences in each pollutant, with lower concentration of PM_2.5_ (*P* < 0.05) and NO_2_ (*P* < 0.01) and reduced intensity of noise (*P* < 0.001) at the green space.

**Table 2:** Distribution of traffic pollutants’ concentrations (µg/m^3^) and noise exposure levels (*L*_Aeq_, dB (A)) from 18:00 to 19:00

	Site	Mean	SD	Min	25%	50%	75%	Max
PM_2.5_*	L	71.67	38.47	46.67	50.00	58.33	73.33	156.67
	H	101.67	53.37	68.33	71.67	88.33	96.67	220.00
NO_2_**	L	52.76	22.46	33.60	34.09	42.56	67.68	94.64
	H	146.03	56.65	92.49	99.53	119.29	203.33	241.04
Noise***	L	44.4	2.0	42.5	42.8	44.0	46.9	47.4
	H	70.1	1.0	68.9	69.1	70.0	71.0	71.7

Nonparametric Mann–Whitney test performed in PM_2.5_; Student’s *t*-test conducted in NO_2_ and noise. L: green space; H: crossroads. *P < 0.05, **P < 0.01, ***P < 0.001

### Developmental conditions

**Fig. 2:**
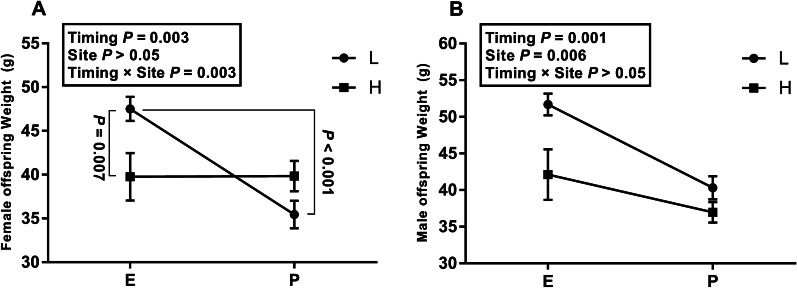
Mean offspring weight (*n* = 10 per group, gram). **A** Female offspring weight and **B** Male offspring weight. L: green space H: crossroads. E: exposure from E0 to E20; P: exposure from PND1 to PND21

No interaction (Fig. 2) was found between exposure site and exposure timing in male offspring’s weight (timing × site *P* > 0.05), and then, main effect analysis excluding the interaction showed H/P rats were lighter than corresponding L/E ones (timing *P* = 0.001, site *P* = 0.006).

There was a, however, significant interaction (Fig. 2) between them over female offspring’s weight (timing × site *P* = 0.003). By single effect analysis, the rats under the exposure from PND1 to PND21 were more prone to low weight compared with those under the exposure from E0 to E20 (12.06 g (6.57, 17.56) *P* < 0.001) at L, and the rats experiencing gestational exposure at L outweighed those at H (7.73 g (2.24, 13.23) *P* = 0.007).

### Epigenetic alterations in *MeCP2*, *Shank3*, H3K4me3 and H3K27me3

**Fig. 3:**
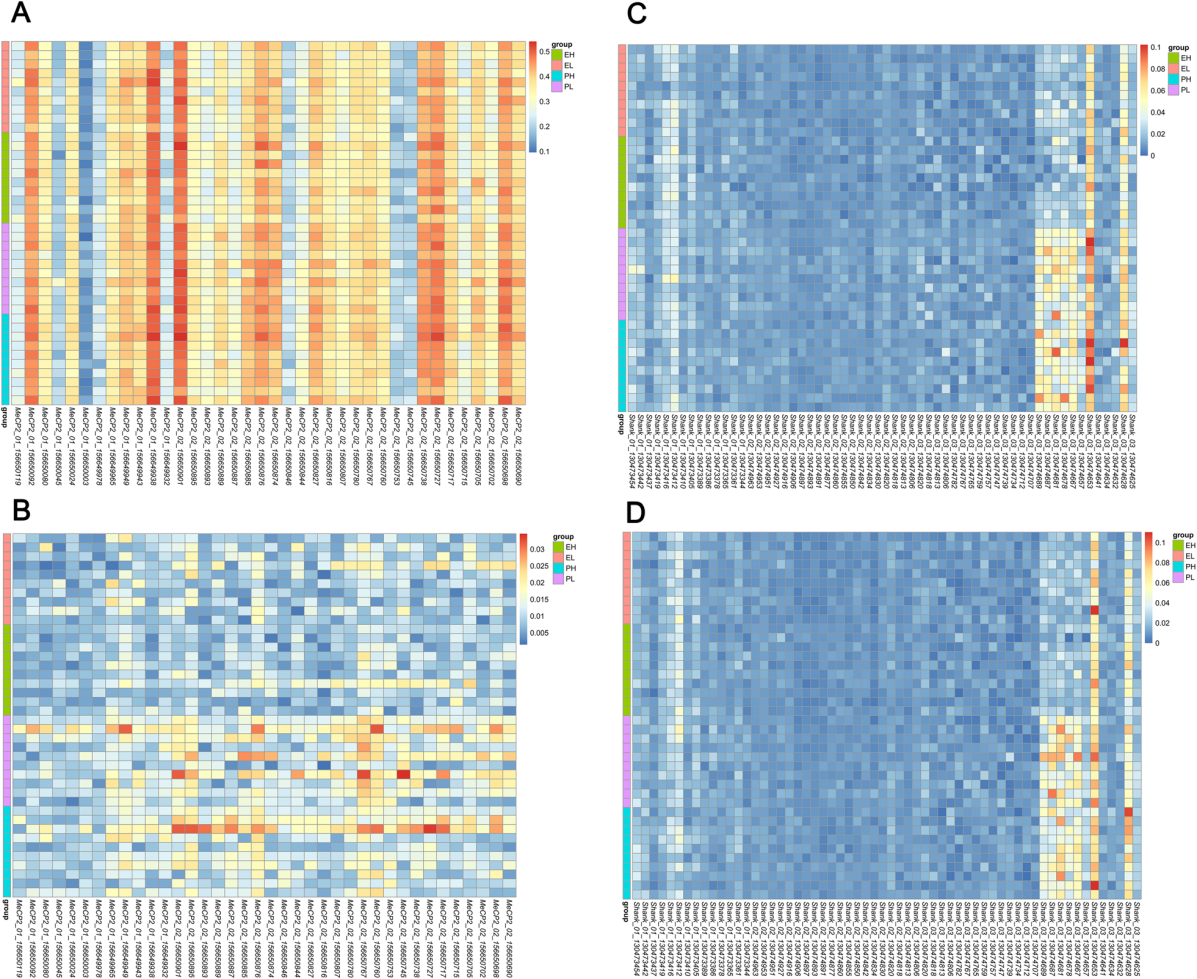
Methylation ratios of each CpG site in *Shank3* and *MeCP2* promoter regions. The methylation ratios of each CpG site were obtained by all read targets divided by the number of gene segments containing methylation

There was no marked interaction found in each of promoter part of *MeCP2* and *Shank3* seen in Table 3. Exposure time, ruling out *Shank3*_01, significantly impacted the ratios of methylation of the two genes in promoter region, with an increase in both gender when the exposure happened in early life compared with one during pregnancy. Moreover, traffic pollution showed a close association with the DNA methylation levels of *Shank3*_02 (*P* < 0.05 in both sexes). The values of every CpG site are pictured in Fig. 3, while the results of statistical analyses are presented in Additional file 1: Table S2.1 and S2.2.

**Table 3:** Average methylation ratios (%) in *Shank3* and *MeCP2* promoter regions (*n* = 10 per group)

	Gender	EL	EH	PL	PH	Timing *P* value	Site *P* value	Timing × site *P* value
*MeCP2_01*	F	31.3 ± 2.0	31.6 ± 1.3	32.3 ± 1.1	32.6 ± 1.2	0.040 (0.037)*	0.481 (0.475)	0.942
*MeCP2_01*	M	0.88 ± 0.17	0.84 ± 0.12	1.21 ± 0.33	1.14 ± 0.25	< 0.001 (< 0.001)***	0.447 (0.441)	0.786
*MeCP2_02*	F	34.2 ± 1.4	34.3 ± 1.7	36.7 ± 1.7	36.4 ± 1.9	< 0.001 (< 0.001)***	0.903 (0.902)	0.662
*MeCP2_02*	M	0.99 ± 0.22	0.97 ± 0.20	1.54 ± 0.38	1.41 ± 0.44	< 0.001 (< 0.001)***	0.456 (0.451)	0.626
*Shank3_01*	F	1.60 ± 0.29	1.74 ± 0.17	1.81 ± 0.19	1.71 ± 0.17	0.199 (0.214)	0.730 (0.739)	0.065
*Shank3_01*	M	1.66 ± 0.23	1.67 ± 0.17	1.81 ± 0.02	1.76 ± 0.31	0.117 (0.113)	0.791 (0.788)	0.705
*Shank3_02*	F	0.93 ± 0.05	0.97 ± 0.06	1.08 ± 0.04	1.14 ± 0.08	< 0.001 (< 0.001)***	0.013 (0.013)*	0.473
*Shank3_02*	M	0.93 ± 0.05	0.99 ± 0.09	1.06 ± 0.08	1.09 ± 0.06	< 0.001 (< 0.001)***	0.041 (0.039)*	0.536
*Shank3_03*	F	1.66 ± 0.13	1.65 ± 0.09	2.48 ± 0.20	2.53 ± 0.20	< 0.001 (< 0.001)***	0.685 (0.682)	0.612
*Shank3_03*	M	1.67 ± 0.11	1.70 ± 0.18	2.43 ± 0.26	2.46 ± 0.06	< 0.001 (< 0.001)***	0.548 (0.543)	0.984

F: female; M: male. L: green space; H: crossroads. E: exposure from embryonic day 0 (E0) to embryonic day 20 (E20); P: exposure from postnatal day (PND) 1 to postnatal day 21. *P < 0.05, **P < 0.01, ***P < 0.001

**Fig. 4:**
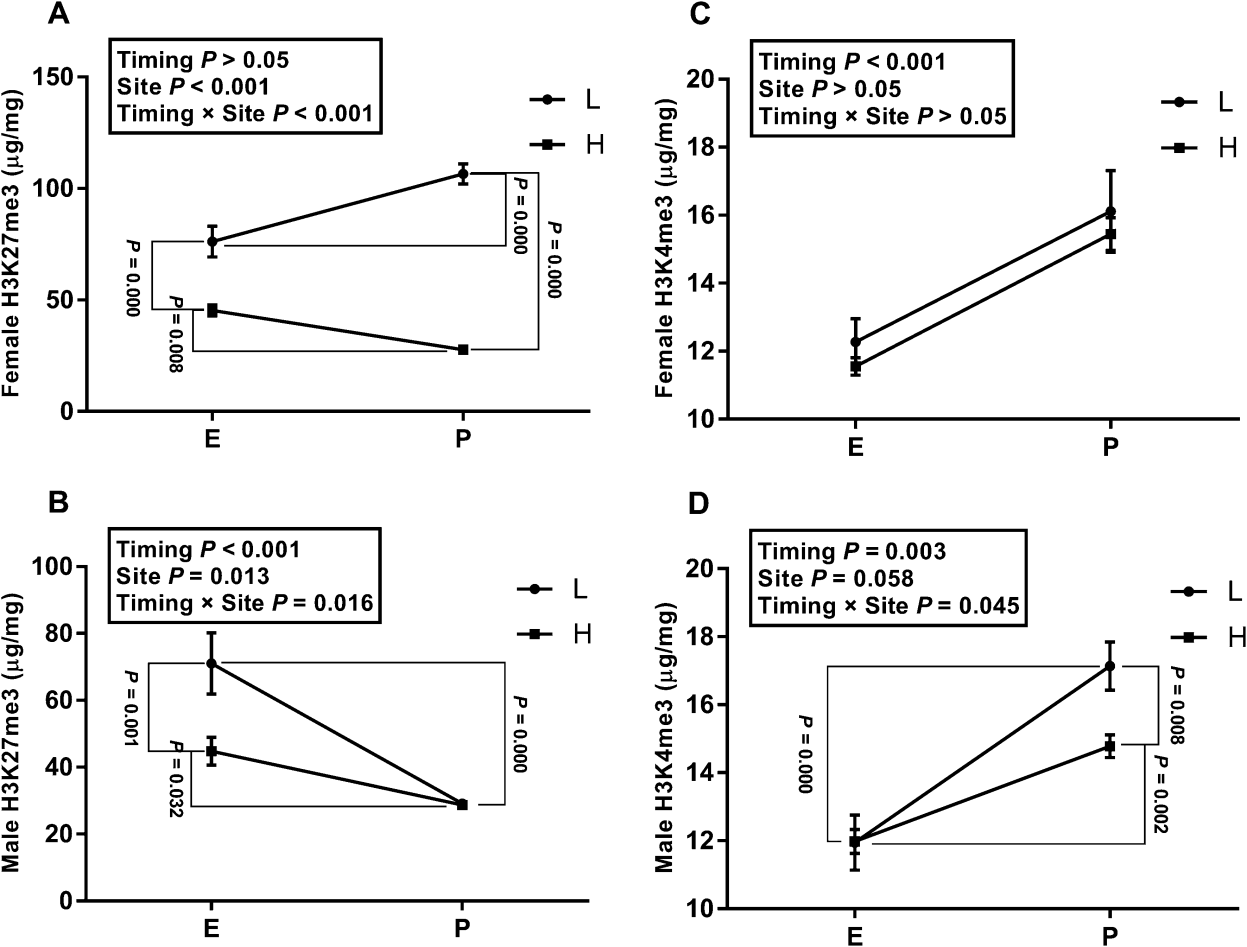
Mean methylation of H3K4me3 and H3K27me3 (*n* = 10 per group). **A** The methylation of H3K27me3 in female rats. **B** The methylation of H3K4me3 in male rats. **C** The methylation of H3K27me3 in female rats. **D** The methylation of H3K4me3 in male rats. L: green space H: crossroads. E: exposure from E0 to E20; P: exposure from PND1 to PND21

Interaction between timing and site was detected regarding the levels of H3K4me3 (timing × site *P* = 0.045) and H3K27me3 (timing × site *P* = 0.016) in male pups. The content of H3K27me3 (Fig. 4B) of the EL group was markedly decreased compared with that of the group of EH or PL (EL: 71.07 µg/mg; EH: 44.76 µg/mg; PL: 29.15 µg/mg, *P* = 0.001, *P* < 0.001, respectively), and there was a robust decline concerning the levels H3K27me3 between the group of EH and PH (EH: 44.76 µg/mg; PH: 28.67 µg/mg, *P* < 0.001). In directly contrast to the pattern of H3K27me3, the male content of H3K4me3 (Fig. 4D) was distinctly different between the group of EL and PL, EH and PH, as well as PL and PH (EL: 11.94 µg/mg; EH: 11.98 µg/mg; PL: 17.14 µg/mg; PH: 14.78 µg/mg, *P* < 0.001, *P* = 0.002, *P* = 0.008, respectively). In female offspring, the amount of H3K4me3 (Fig. 4C) of rats under postnatal exposure was, without interaction between them, higher than those under prenatal exposure (timing *P* < 0.001). Intriguingly, the content of H3K27me3 (Fig. 4A) showed an opposite trend in females that there was an increase for the PL group compared with the EL group (EL: 76.16 µg/mg; PL: 106.61 µg/mg, *P* < 0.001), while a significant drop for the PH group compared with the EH group (EH: 45.30 µg/mg; PH: 27.71 µg/mg, *P* = 0.008).

### The content of MeCP2 and Shank3

**Fig. 5:**
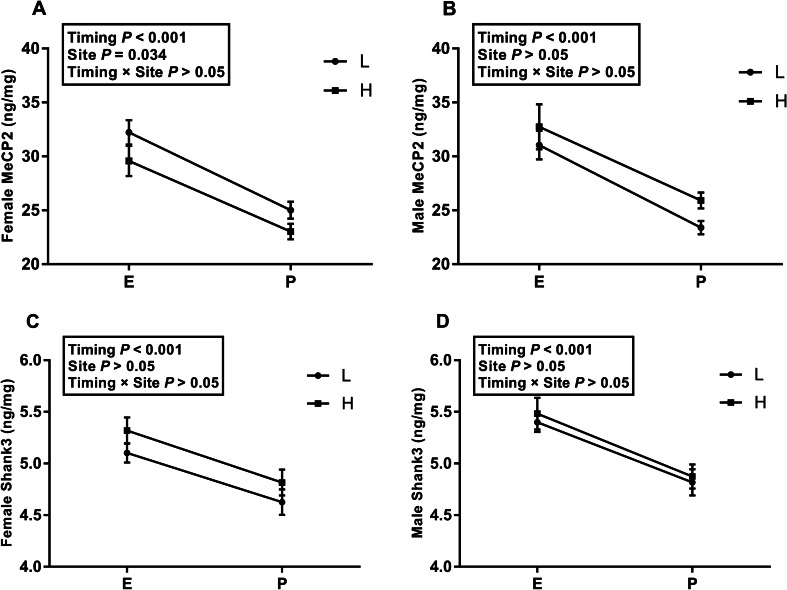
Mean protein expression of MeCP2 and Shank3 (*n* = 10 per group). **A** The mean protein expression of MeCP2 in female rats. **B** The mean protein expression of MeCP2 in male rats. **C** The mean protein expression of Shank3 in female rats. **D** The mean protein expression of Shank3 in male rats. L: green space H: crossroads. E: exposure from E0 to E20; P: exposure from PND1 to PND21

A significant increase in *MeCP2* and *Shank3* promoter methylation in frontal cortex was correlated with a reduction in MeCP2 and Shank3 levels (Fig. 5). With no interaction detected between exposure site and timing, female offspring under early-life exposure contain less MeCP2 (Fig. 5A, EL: 32.23 ng/mg; EH: 29.58 ng/mg; PL: 25.01 ng/mg; PH: 23.03 ng/mg, timing *P* < 0.001; site *P* = 0.034) and Shank3 (Fig. 5C, EL: 5.10 ng/mg; EH: 5.31 ng/mg; PL: 4.63 ng/mg; PH: 4.82 ng/mg, timing *P* < 0.001; site *P* > 0.05) compared to those under maternal one, whose trend of the protein expression of *MeCP2* (Fig. 5B, EL: 31.05 ng/mg; EH: 32.75 ng/mg; PL: 23.40 ng/mg; PH: 25.91 ng/mg, timing *P* < 0.001; site *P* > 0.05) and *Shank3* (Fig. 5D, EL: 5.40 ng/mg; EH: 5.48 ng/mg; PL: 4.82 ng/mg; PH: 4.87 ng/mg, timing *P* < 0.001; site *P* > 0.05) is the same in males.

### Social behavior: three-chamber social test

**Fig. 6:**
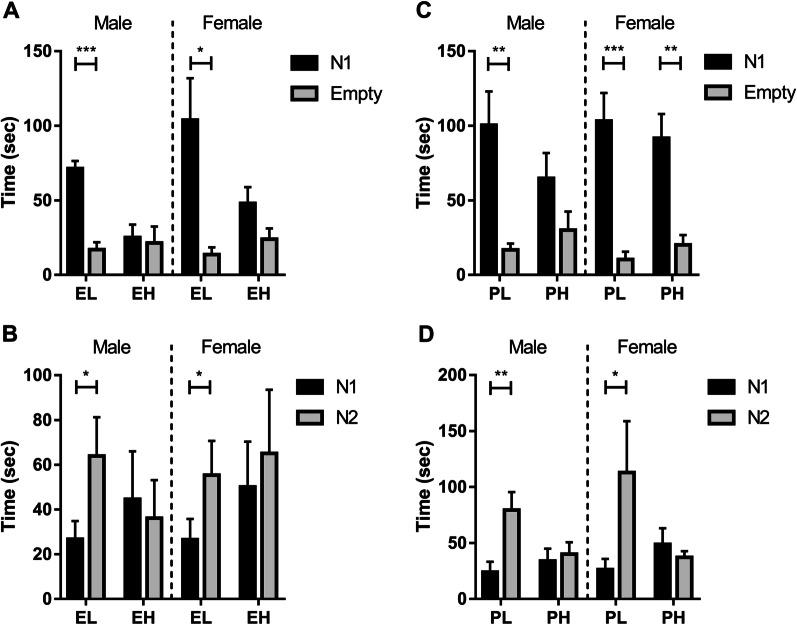
Three-chamber social test (*n* = 10 per group). Cumulative time spent in the chamber containing a novel rat (N1) and one without it was determined to assess sociability (**A**, **C**). In the social novelty phase (**B**, **D**), this ability was judged by cumulative time spent in the chambers holding a unfamiliar rat (N2) and the previous one (N1), respectively. L: green space H: crossroads. E: exposure from E0 to E20; P: exposure from PND1 to PND21. Student’s *t*-test conducted during all testing. *P < 0.05, **P < 0.01, ***P < 0.001.

In the sociability phase (Fig. 6A, C), the females and males exposed to traffic pollution displayed a palpable preference for contacting a novel one (N1) rather than an empty cylinder. The time that the groups under traffic pollutants exposure spent in two end compartments exhibited no significant differences except for the female group of PH. In the social novelty phase (Fig. 6B, D), test rats were supposed to prefer to interact with the newcomer (N2), which was observed in the rats at L, though not at H.

## Discussion

In the present study, the rats exposed at a crossroad or a green space for either full gestation or three weeks after birth showed significant differences in development, the levels of methylation and corresponding protein expression, and behavior.

In addition to traffic-related air pollution, noise as a both physical and psychological stress is also taken into account in the study [28]. Different kinds of maternal and early-life stress can regulate epigenetics, in particular DNA methylation, which may regulate gene expression and in turn affect stress-related hormones, such as cortisol and placental corticotropin-releasing hormone (CRH) and therefore may influence the newborn’s cognitive development [29, 30]. In mice, chronic traffic noise stress causes DNA methylation changes in hippocampus and inferior colliculus [31], and a reduction in brain volume, cortical thickness and hippocampal volume with poor performance in novel object recognition test and the Morris water task [32]. Thus, noise and air pollution could have a joint effect on the onset of neurological disorders.

Increasing epidemiologic studies have found an association between air pollution exposure during pregnancy and low birth weight [33]. Among them, a study investigating London’s road traffic air and noise pollution suggested that PM_2.5_ was adversely affecting fetal growth and the impact of road traffic noise on birth weight outcomes was little after adjusted for primary traffic-related air pollutants [34]. In animal studies, Guo and her colleagues, however, reported that long-term environmental noise exposure was linked with decreased body weight gain in developing rats [31]. Moreover, the mice exposed from E0 to PND21 to 250–300 µg/m^3^ diesel exhaust were in similar size on PND21 [10]. Taken together, the result is inconsistent. We found real-world traffic pollution exposure and exposure timing both led to aberrant weight of 25-day rats, which might be caused by the lack of the protection of placental barrier and the effects of traffic-related air pollutants and noise, such as oxidative stress [35].

We found both male and female offspring under early-life exposure had marked higher levels of methylation at the *MeCP2* promoter and the less content of MeCP2 when compared with those under exposure during full gestation, which implied postnatal time was a sensitive window for perturbations of methylation. The same result was also observed in the frontal cortex of ASD patients and in the mice that was modified on at the ~ 500-bp TSS region of *MeCP2* with a range of behavioral alterations, such as reduced social interaction [21, 36]. In the prenatal period, MeCP2 acts on ADAM10/NOTCH signaling via miR-197 to disrupt the differentiation of neural progenitor cells into neurons [37]. After birth, the aberrant protein transcriptionally inhibits the expression of GluA2 and then influences homeostatic plasticity, a critical form of synaptic plasticity [38]. Also, the deficiency of MeCP2 reduces the strength of excitatory synaptic input [39, 40]. Taken together, MeCP2 is associated with normal synaptic functions, and deviations from which is a possible cause leading to ASD. Moreover, *MeCP2* Serine 421 site regulates *BDNF* gene expression to adjust neural activity, and abnormalities in BDNF level may underlie the pathology of ASD [41].

We observed the same change in *Shank3* methylation and protein expression like *MeCP2*. In addition, our data clearly showed epigenetic dysregulation of *Shank3* promoter region after TSS, which was associated with traffic pollution exposure. Our findings correspond to previous studies.

A study reported an increase in *Shank3* methylation in the intragenic CpG islands-2, 3 and 4 in postmortem brain tissues from 54 ASD patients [42]. Li and his colleagues found the rats exposed to high dose of PM_2.5_ from PND8 to PND22 had significantly decreased mRNA level and protein expression of *Shank3* [43]. The synaptic proteins directly contribute to the formation of dendritic spine that forms the postsynaptic part of most excitatory synapses via remodeling of actin cytoskeleton, and synaptic connections that are necessary for constructing functional neural circuits, which is proposed as the mechanism of neuronal activities, such as learning and memory [15, 38]. Sacai has demonstrated that reduced excitatory synaptic transmission can lead to impaired social interaction and mild vocalization abnormality in pyramidal neurons of mouse prefrontal cortex [44]. Abnormalities in the number and shape of dendritic spines have been observed in ASD cases, and the consequences on behavioral changes are the main features of ASD.

Generally, we observed increased H3K4me3 and reduced H3K27me3 in the brains of the PH/PL rats compared to corresponding those in EH/EL groups. A previous study found higher H3K4me3 levels in bronchial epithelial cells both from normal and chronic obstructive pulmonary disease (COPD)-diseased subjects who repeatedly exposed to air pollution [45]. Global levels of H3K27me3 of truck drivers were lower than that of office workers in blood leukocytes [46]. Moreover, reduced H3K27me3 in brains can lead to abnormal expression of the *Hox* genes, which ultimately resulted in neural tube defects [47]. The decreased H3K27me3 and elevated H3K4me3 were shown in the promoter of *Engrailed-2* (EN-2) that is a candidate gene of ASD with the observed EN-2 overexpression [48, 49]. In the context of its overexpression, GABA cell morphology was impaired, and spine density and the number of mature synapses were reduced [50, 51]. Intriguingly, the levels of H3K27me3 of the female group showed an inverse trend regarding the factor of exposure timing. Nugent and his team demonstrated that O-linked N-acetylglucosamine transferase (OGT) regulated the H3K27me3 at a high level in the female-specific placenta to create resilience to environmental risks for neurodevelopmental vulnerability [52]. Thus, excess H3K27me3 is a protective mechanism against neonatal risks for females, but it can be disrupted by traffic pollution.

Rats at the green space showed obvious sociability preference and social novelty, but those at the crossroad was lack of it. Our findings correspond with that of previous studies reporting reduced social novelty preference via testing nose to nose sniff rates in male adult mice that were subjected to developmental exposure to air pollution [53], and abnormal social novelty via three-chamber social test in female adult mice that experienced diesel exhaust exposure from E0 to PND21 [10]. Moreover, adverse effects of traffic-related air pollution and noise on cognitive function have been reported [54, 55].

There are some limitations in our study. The concentration of PM_2.5_ and NO_2_ at the green space was comparatively high compared with some epidemiological studies conducted in Europe, and the pollutants at the crossroad were relatively low despite prolonged exposure days per week compared with other animal experiments, which may partly attenuated the differences of epigenetic perturbations. Although we have used the same exposure time for different groups, there are still unknown confounders existing in the real-world experiment. Furthermore, our study cannot distinguish the impact of traffic-related air pollution from that of traffic noise, and thus, the main environmental insult is still elusive. The small number of samples can lead to inconsistent interaction between exposure timing and exposure site. In terms of the dynamic of methylation, we conducted only a behavioral experiment to determine whether or not traffic pollution induces ASD-related behaviors.

## Conclusions

The present study explicitly provides evidence about the impact induced by traffic pollution and high sensitivity after birth on offspring weight, epigenetic regulation, protein expression and social behaviors. It also urges reducing road traffic air and noise pollution and neonatal protective measures taken to against environmental risks for public health.

## Supplementary Information

**Additional file 1.Table S1.** Primers used for the MethylTargetTM assays. **Table S2.1.** Analyses of MeCP2 methylation of each CpG site in promoter regions (n = 10). **Table S2.2.** Analyses of Shank3 methylation of each CpG site in promoter regions (n = 10).

## Data Availability

The datasets used and/or analyzed during the current study are available from the corresponding author on reasonable request.

## Abbreviations

ASD: Autistic spectrum disorder
NO_2_: Nitrogen dioxide
PM_2.5_: Particulate matter with aerodynamic diameter < 2.5 µm
BMI: Body mass index
HPTMs: Histone posttranslational modifications
*MeCP2*: Methyl-CpG binding protein 2
*Shank3*: SH3 and multiple ankyrin repeat domains 3
H3K4me3: Tri-methylation of lysine 4 on histone H3
H3K27me3: Tri-methylation of lysine 27 on histone H3
PND: Postnatal day
