# Supplementary material for: Prenatal and postnatal traffic pollution exposure, DNA methylation in Shank3 and MeCP2 promoter regions, H3K4me3 and H3K27me3 and sociability in rats’ offspring
Source: Clin Epigenetics. 2021 Sep 26;13:180. doi: 10.1186/s13148-021-01170-x (PMC8474908; doi:10.1186/s13148-021-01170-x)
Supplement: Supplementary file 1 — Additional file 1.Table S1. Primers used for the MethylTargetTM assays. Table S2.1. Analyses of MeCP2 methylation of each CpG site in promoter regions (n = 10). Table S2.2. Analyses of Shank3 methylation of each CpG site in promoter regions (n = 10). [file 13148_2021_1170_MOESM1_ESM.docx]

Table S1 Primers used for the MethylTarget^TM^ assays

|  | Sequence |
| --- | --- |
| *MeCP2_01* Forward | AAAGGGGYGGATTTTATTTAGTTG |
| *MeCP2_01* Reverse | CCTCAACAAACAACTTTACTACTTCTACA |
| *MeCP2_02* Forward | GGGGATAGYGYGATTTAGGGAGAG |
| *MeCP2_02* Reverse | CCRATAATAACTTTCTCCACTTATCTCC |
| *Shank3_01* Forward | GGGAGGGGGTGAYGAGTG |
| *Shank3_01* Reverse | ACCRAAATAAACCCTACTAAAAATAAAACA |
| *Shank3_02* Forward | GGGAGGGAGGTTGGAATAG |
| *Shank3_02* Reverse | CCCAACTAACCCTCCAAAAC |
| *Shank3_03* Forward | TTYGGATTTGYGGGYGTAGGGTAT |
| *Shank3_03* Reverse | TACCCCTATATAACCCRCTAACAATAACC |

Table S2.1 Analyses of *MeCP2* methylation of each CpG site in promoter regions (*n* = 10)

| Variable | Position | Timing | Site | Timing × Site | Timing † | Site † |
| --- | --- | --- | --- | --- | --- | --- |
| *MeCP2_01* | 156650119 | ^#^ |  |  | ^#^ |  |
| *MeCP2_01* | 156650092 | ^#^ |  |  | ^#^ |  |
| *MeCP2_01* | 156650080 | ^##^ |  |  | ^##^ |  |
| *MeCP2_01* | 156650045 |  |  |  |  |  |
| *MeCP2_01* | 156650024 | ^*^ |  |  | ^*^ |  |
| *MeCP2_01* | 156650003 | ^###^ |  |  | ^###^ |  |
| *MeCP2_01* | 156649978 | ^#^ |  |  | ^#^ |  |
| *MeCP2_01* | 156649965 | ^#^ |  |  | ^#^ |  |
| *MeCP2_01* | 156649949 | ^#^ |  |  | ^#^ |  |
| *MeCP2_01* | 156649943 | ^##^ |  |  | ^##^ |  |
| *MeCP2_01* | 156649938 | ^###^ |  |  | ^###^ |  |
| *MeCP2_01* | 156649932 | ^##^ |  |  | ^##^ |  |
| *MeCP2_02* | 156650901 | ^*###^ |  |  | ^*###^ |  |
| *MeCP2_02* | 156650895 | ^***###^ |  |  | ^***###^ |  |
| *MeCP2_02* | 156650893 | ^**###^ |  |  | ^**###^ |  |
| *MeCP2_02* | 156650889 | ^*#^ |  |  | ^*#^ |  |
| *MeCP2_02* | 156650887 | ^**##^ | ^##^ |  | ^**##^ | ^##^ |
| *MeCP2_02* | 156650885 | ^***##^ |  |  | ^***##^ |  |
| *MeCP2_02* | 156650876 | ^*###^ |  |  | ^*###^ |  |
| *MeCP2_02* | 156650874 | ^**#^ |  |  | ^**#^ |  |
| *MeCP2_02* | 156650846 | ^*###^ |  |  | ^*###^ |  |
| *MeCP2_02* | 156650844 | ^**^ |  |  | ^**^ |  |
| *MeCP2_02* | 156650827 | ^**###^ |  |  | ^**###^ |  |
| *MeCP2_02* | 156650816 | ^**##^ |  |  | ^***##^ |  |
| *MeCP2_02* | 156650807 | ^*##^ |  | ^#^ | ^*^ |  |
| *MeCP2_02* | 156650780 | ^*##^ |  |  | ^*###^ |  |
| *MeCP2_02* | 156650767 | ^**###^ |  | ^#^ | ^**^ |  |
| *MeCP2_02* | 156650760 | ^**###^ |  |  | ^**###^ |  |
| *MeCP2_02* | 156650753 | ^*###^ |  |  | ^*###^ |  |
| *MeCP2_02* | 156650745 | ^#^ |  |  | ^#^ |  |
| *MeCP2_02* | 156650738 | ^**##^ |  |  | ^**##^ |  |
| *MeCP2_02* | 156650727 | ^**#^ |  |  | ^**#^ |  |
| *MeCP2_02* | 156650717 | ^**#^ |  |  | ^**#^ |  |
| *MeCP2_02* | 156650715 | ^**^ |  |  | ^**^ |  |
| *MeCP2_02* | 156650705 | ^*^ |  |  | ^*^ |  |
| *MeCP2_02* | 156650702 | ^**##^ | ^#^ |  | ^***##^ | ^#^ |
| *MeCP2_02* | 156650698 | ^**#^ |  |  | ^**#^ |  |
| *MeCP2_02* | 156650690 | ^*#^ |  |  | ^*#^ |  |

Analysis of variance (ANOVA) with a factorial design was performed. †: adjusted model that is without interaction between timings and sites is applied. ^*^*P* < 0.05, ^**^*P* < 0.01, ^***^*P* < 0.001 in females. ^#^*P* < 0.05, ^##^*P* < 0.01, ^###^*P* < 0.001 in males.

Table S2.2 Analyses of *Shank3* methylation of each CpG site in promoter regions (*n* = 10)

| Variable | Position | Timing | Site | Timing × Site | Timing † | Site † |
| --- | --- | --- | --- | --- | --- | --- |
| *Shank3_01* | 130473454 | ^**#^ |  |  | ^**#^ |  |
| *Shank3_01* | 130473442 | ^*#^ | ^#^ |  | ^*#^ | ^#^ |
| *Shank3_01* | 130473437 | ^#^ |  |  | ^#^ |  |
| *Shank3_01* | 130473419 |  |  |  |  |  |
| *Shank3_01* | 130473416 |  |  | ^**^ |  |  |
| *Shank3_01* | 130473412 |  |  |  |  |  |
| *Shank3_01* | 130473410 |  |  |  |  |  |
| *Shank3_01* | 130473405 |  |  |  |  |  |
| *Shank3_01* | 130473389 |  |  |  |  |  |
| *Shank3_01* | 130473386 |  | ^*^ | ^#^ |  | ^*^ |
| *Shank3_01* | 130473378 |  |  |  |  |  |
| *Shank3_01* | 130473365 |  |  |  |  |  |
| *Shank3_01* | 130473361 |  |  |  |  |  |
| *Shank3_01* | 130473344 |  |  | ^*^ |  |  |
| *Shank3_02* | 130474963 | ^**^ |  |  | ^**^ |  |
| *Shank3_02* | 130474953 |  | ^*^ |  |  | ^*^ |
| *Shank3_02* | 130474951 | ^**^ |  |  | ^**^ |  |
| *Shank3_02* | 130474927 |  |  |  |  |  |
| *Shank3_02* | 130474916 | ^***##^ |  |  | ^***##^ |  |
| *Shank3_02* | 130474906 | ^#^ | ^##^ |  | ^#^ | ^##^ |
| *Shank3_02* | 130474897 | ^*##^ |  |  | ^*##^ |  |
| *Shank3_02* | 130474893 | ^##^ | ^##^ |  | ^##^ | ^##^ |
| *Shank3_02* | 130474891 | ^*^ |  | ^#^ | ^*^ |  |
| *Shank3_02* | 130474877 |  |  | ^#^ |  |  |
| *Shank3_02* | 130474860 | ^**^ |  | ^**^ |  |  |
| *Shank3_02* | 130474855 | ^**^ |  |  | ^**^ |  |
| *Shank3_02* | 130474850 |  |  |  |  |  |
| *Shank3_02* | 130474842 | ^**^ |  |  | ^**^ |  |
| *Shank3_02* | 130474834 | ^##^ |  |  | ^##^ |  |
| *Shank3_02* | 130474830 |  |  |  |  |  |
| *Shank3_02* | 130474820 | ^**#^ |  |  | ^**#^ |  |
| *Shank3_02* | 130474818 |  |  |  |  |  |
| *Shank3_02* | 130474813 | ^**^ | ^**^ |  | ^**^ | ^**^ |
| *Shank3_02* | 130474806 | ^**^ |  | ^*^ |  |  |
| *Shank3_03* | 130474820 | ^*^ | ^*^ |  | ^*^ | ^*^ |
| *Shank3_03* | 130474818 |  |  |  |  |  |
| *Shank3_03* | 130474813 |  |  |  |  |  |
| *Shank3_03* | 130474806 |  |  |  |  |  |
| *Shank3_03* | 130474782 |  |  |  |  |  |
| *Shank3_03* | 130474767 |  | ^*^ |  |  | ^*^ |
| *Shank3_03* | 130474765 |  |  |  |  |  |
| *Shank3_03* | 130474759 | ^*^ | ^*#^ |  | ^*^ | ^*^ |
| *Shank3_03* | 130474757 |  |  |  |  |  |
| *Shank3_03* | 130474747 |  |  |  |  |  |
| *Shank3_03* | 130474739 |  |  |  |  |  |
| *Shank3_03* | 130474734 |  |  |  |  |  |
| *Shank3_03* | 130474712 |  |  |  |  |  |
| *Shank3_03* | 130474707 |  |  |  |  |  |
| *Shank3_03* | 130474689 | ^***###^ |  |  | ^***###^ |  |
| *Shank3_03* | 130474687 | ^***###^ |  |  | ^***###^ |  |
| *Shank3_03* | 130474681 | ^***###^ |  |  | ^***###^ |  |
| *Shank3_03* | 130474678 | ^***###^ |  |  | ^***###^ |  |
| *Shank3_03* | 130474667 | ^***###^ |  |  | ^***###^ |  |
| *Shank3_03* | 130474657 | ^***##^ |  |  | ^***##^ |  |
| *Shank3_03* | 130474653 | ^***^ |  |  | ^***^ |  |
| *Shank3_03* | 130474641 |  |  |  |  |  |
| *Shank3_03* | 130474634 |  |  | ^#^ |  |  |
| *Shank3_03* | 130474632 | ^**^ |  |  | ^**^ |  |
| *Shank3_03* | 130474628 | ^***##^ | ^#^ |  | ^***#^ | ^#^ |
| *Shank3_03* | 130474625 | ^**##^ |  |  | ^**##^ |  |

Analysis of variance (ANOVA) with a factorial design was performed. †: adjusted model that is without interaction between timings and sites is applied. ^*^*P* < 0.05, ^**^*P* < 0.01, ^***^*P* < 0.001 in females. ^#^*P* < 0.05, ^##^*P* < 0.01, ^###^*P* < 0.001 in males.
